# Oculomotor Neurocircuitry, a Structural Connectivity Study of Infantile Nystagmus Syndrome

**DOI:** 10.1371/journal.pone.0125380

**Published:** 2015-04-10

**Authors:** Nasser H. Kashou, Angelica R. Zampini

**Affiliations:** BioMedical Imaging Lab, Wright State University, Dayton, OH, United States of America; University of Jaén, SPAIN

## Abstract

Infantile Nystagmus Syndrome (INS) is one of the leading causes of significant vision loss in children and affects about 1 in 1000 to 6000 births. In the present study, we are the first to investigate the structural pathways of patients and controls using diffusion tensor imaging (DTI). Specifically, three female INS patients from the same family were scanned, two sisters and a mother. Six regions of interest (ROIs) were created manually to analyze the number of tracks. Additionally, three ROI masks were analyzed using TBSS (Tract-Based Spatial Statistics). The number of fiber tracks was reduced in INS subjects, compared to normal subjects, by 15.9%, 13.9%, 9.2%, 18.6%, 5.3%, and 2.5% for the pons, cerebellum (right and left), brainstem, cerebrum, and thalamus. Furthermore, TBSS results indicated that the fractional anisotropy (FA) values for the patients were lower in the superior ventral aspects of the pons of the brainstem than in those of the controls. We have identified some brain regions that may be actively involved in INS. These novel findings would be beneficial to the neuroimaging clinical and research community as they will give them new direction in further pursuing neurological studies related to oculomotor function and provide a rational approach to studying INS.

## Introduction

Periodic, alternating involuntary eye movements detected within the first six months of life characterize Infantile Nystagmus Syndrome (INS). INS is one of the leading causes of significant vision loss in children and affects about 1 in 1000 to 6000 births [1]. INS accounts for about 2–8% of children with visual impairment or legal blindness who utilize services for the visually impaired. About 40% of INS patients have an autosomal dominant inheritance pattern [2] thus it is important to note that a few studies have alluded to the possible genetic predisposition associated with several genes: FRMD7 (Xq26-27) [3], Xq28 [4], NYS1 (Xp11.4-p11.3) [5], NYS2 (6p12) [6], while others have found no link with INS and specifically 6p12, 7p11, and 15q11 [7]. While isolating a gene is still being researched, this is not the objective of this paper. In general, nystagmus in early life may be associated with any of these genes and can be diagnosed as INS (idiopathic) or associated with sensory system diseases such as albinism, retinal degeneration and optic nerve hypoplasia. In order to know if a patient has INS without these associated diseases, several tests need to be utilized including reviewing patient history, neuro-ophthalmological exam, eye movement recordings, brain imaging as well as electrophysiological testing. INS is characterized as an accelerating velocity exponential slow phase jerk nystagmus; a high velocity (saccadic) eye movement moves the eyes onto a target with a subsequent slow but accelerating eye movement (pursuit) away from the target. These INS eye movements can further be divided into more than 12 types [8]. Hence INS consists of a heterogeneous patient population. Several suggestions have been brought forth in regards to the heterogeneous origins of INS. Some have associated it with a dysfunction in the pursuit portion of the eye movement causing pendular and pseudopendular waveforms [9–11]. Smooth pursuit uses negative feedback to prevent retinal slip concentrating image stabilization on or near the fovea. The flocculus of the vestibulocerebellum is vital for ocular stabilization thus unwanted nystagmus is not suppressed with floccular lesions [12]. Others have suggested it is the saccadic portion that is culprit [13]. Lesions of the dorsal vermis of the cerebellum changes accuracy, latency, trajectory and dynamic properties of rapid jerk eye movements. Inhibitory burst neurons (IBN) block the saccade by strongly inhibiting the abducens nucleus, which drives the muscle of the saccade [14]. Responsible for linear head-eye coordination, the vestibuloocular reflex maintains a stable gaze in space [12]. There needs to be a constant balancing between the push-pull pair in each semicircular canal; otherwise, a persistent nystagmus occurs. Rotational coordination is controlled by the optokinetic system, which supplements the vestibular system. Commonly found at infancy, congenital nystagmus (CN) and manifest latent nystagmus (MLN) possess anomalies of the smooth pursuit, fixation and optokinetic systems due to unknown neuronal dis-connectivity wherein the slow phase waveforms of CN have an increased exponential velocity while MLN is a decreasing linear velocity [15]. Due to inconclusive results, another group believes that normal oculomotor function cannot be used to fully understand all the waveforms possible in INS [16]. For instance, an INS patient may be albino with a cerebellar disorder thus concluding there are a variety of mechanisms underlying nystagmus features. One key feature of INS is the “null zone”, which exists in about 70% of patients and is a direction of gaze where the INS eye movement is minimized in terms of frequency and amplitude and at times may become negligible [2]. The majority of patients with a null zone experience it within 10 degrees of the primary, straight-ahead, position, resulting in increased fixation duration which allows visual acuity of 20/70 or better for these patients. In general, the longer the foveation time on the target the better the visual acuity [2, 17–26]. While much is known about INS in terms of eye movements and associated sensory system disease, the cause of INS as well as the generator site(s) of INS remains elusive [27].

Current therapeutic interventions are limited for patients with INS; there is no cure. Certain types of eye muscle surgery may damp the intensity of the nystagmus, allow improved foveation time and some improvement in visual acuity [24, 27, 28]. Pharmacologic intervention has been of some benefit for acquired nystagmus but of very limited benefit in INS [29]. Biofeedback, utilizing auditory feedback, has been shown to decrease the intensity of the nystagmus; unfortunately, no significant change in binocular visual acuity was reported [30]. Biofeedback training contributed to control of the eye jerking; however, interestingly, no increase in visual acuity was documented even with an increased foveation period [31]. Contact lenses have also been shown to damp the nystagmus in some INS patients, allowing for some improvement in visual acuity; yet, contacts work only in a fraction of patients [20]. Contact lenses initially reduce the nystagmus, but this improvement is diminished in few months once the eyes adapt and nystagmus returns to its previous state. Base-out prisms can be used to capitalize on the damping of the nystagmus with convergence and improve vision in some INS patients. Voluntary vergence impacts the amplitude and frequency of nystagmus [32]. With a desired level of acuity to perform a specific task, patients may have nystagmus blockage syndrome resulting in strabismus, which may cause horizontal dissociated divergence [31].

The types of abnormal eye movements that characterize INS are well known, however little is known of the structural and functional correlates of INS; that is, the brain sites that generate the abnormal eye movements. No animal lesion studies or human clinical studies have uncovered the structural correlates of the INS. However, oculomotor function is known to involve the brainstem and cerebellum as well as the thalamus, basal ganglia and cortical regions. Eye fields contain sub-regions within these cortical regions of the brain that project onto the oculomotor regions of the brainstem. The dorsolateral prefrontal cortex (DLPFC) has executive control over eye movements. Connected to the DLPFC is the frontal and supplementary eye fields generating and sequencing saccades voluntarily. The saccadic stimulations are then projected onto the parietal eye fields converting the sensory stimuli to consequential motor commands. Receiving the converging inputs from these cortical regions and basal ganglia, the superior colliculus projects the stimuli to the paramedian pontine reticular formation (PPRF) of the brainstem to command movements. Simultaneously, the PPRF projects the stimuli to the thalamus to access the cortex and basal ganglia. The PPRF is a saccadic burst generator protruding to eye muscle motoneurons. The omnipause neurons (OPNs) inhibit these eye movements by directing preventing projection onto bust neurons (BNs). Within the basal ganglia, responsible for regulating movement, the caudate, putamen and globus pallidus act upon the motor thalamus receiving motor stimuli from the cortex. The direct pathway of the oculomotor loop stimulates motor function from corresponding eye fields to the caudate and putamen. Triggering the substantia nigra inhibits the superior colliculus (SC) thus preventing unwanted saccadic eye movements [33]. When a saccade is desired, the caudate and putamen inhibit the substantia nigra pars reticulata to release the SC and generate the saccade [34]. The regulation of these oculomotor movements is the substantia nigra pars compacta. The strongest of the nerve pathways connecting eyes to the brain in humans and many other mammals involve the lateral geniculate nucleus (LGN) of the dorsal thalamus and the SC [35]. M-cells (magnocellular) are the deepest layer of ganglion cells of the LGN specialized to detect eye motion [36]. Further experimental evidence reveals a corollary discharge oculomotor pathway from brainstem to frontal eye field via the thalamus [33, 34].

In the cerebellum, the oculomotor vermis, paravermis lobules (V, IV, and VII), the uvula and nodulus, flocculus, and ventral paraflocculus all receive sensory information relevant to eye movements from the brainstem and cerebrum [37]. Flocculus and paraflocculus subregions discharge in relation to head motion or visual target motion which initiates vestibuloocular reflex, vergence and smooth pursuit movements. These discharges act as a neural integrator controlling the brainstem circuits that convert velocity commands into position commands for eye movements [14]. Specifically, neurons in paramedian tracts, MVN and nucleus prepositus hypoglossi are the horizontal oculomotor integrators while the oculomotor integrators for vertical and torsional eye position commands involve the nucleus prepositus hypoglossi, superior vestibular nucleus and the interstitial nucleus of Cajal in the midbrain [14]. Experimental evidence shows lesions in the flocculus and paraflocculus are caused by asymmetry of Purkinje fibers resulting in downbeat nystagmus [38]. The nodulus and uvula has shown to control smooth pursuit, optokinetic nystagmus and low frequency vestibuloocular reflexes; however, dysfunction arises when there is an inability to control spatial orientation [39]. Complete removal of the uvula and nodulus in rhesus monkeys has proven a loss of spatial orientation within the vestibular nuclei. However, regardless of orientation the otolith-canal suppresses downbeat nystagmus and regulates the tilt-translation by signaling linear acceleration [40]. Concluding whether this is a visual or oculomotor dysfunction is yet to be determined. Furthermore, the dorsal vermis specifically controls saccades; likewise, lesions will change accuracy, latency, trajectory and dynamic properties [14]. It is known the suppression of unwanted saccades is accomplished by activating inhibitory burst neurons (IBN) choking the abducens nucleus to direct the saccadic muscle.

In humans, a total of about 40,000,000 fibers that arise from both cerebral hemispheres terminate on the pontine neurons. Which, in turn, send almost half as many fibers to the cerebellum [41]. The oculomotor regions of the cortex project to pontine nuclei (PN). Specifically, almost all terminations are found in the dorsal portion of the PN [42]. Within the cerebral cortex, the corticopontine fibers project to the pontine grey nuclei, which utilize the middle cerebellar penduncle to connect the pontocerebellar fibers to the cerebellum. The cortico-ponto-cerebellar pathway is a vital route for communication between the cortex of the cerebrum and cerebellum. Information received in the cerebellar vermis initiates and executes motor commands [43].

Animal studies have shown that the pons receives oculomotor input from other brainstem and cerebral regions, which then project to the cerebellum. The target of the axons of the pontine projection neurons is the cerebellum [44] where the posterior vermis was identified as an oculomotor region [45]. More specifically, lobules VI and VII in the vermis were reported as receiving oculomotor input from the pons [46, 47]. These areas, along with the dorsal paraflocculus, receive input from the PN in the pons which receive input from the cerebellar cortex [42]. Recently, using functional methods such as functional magnetic resonance imaging (fMRI), we have been able to isolate these regions for normal oculomotor control and on other types of nystagmus in the cerebrum, cerebellum and some brainstem [27, 48]. Based on these studies, we hypothesized that the cerebellar brainstem regions, specifically the pons, will be affected in patients with INS (compared to controls).

## Methods

### Subjects

**Fig 1:**
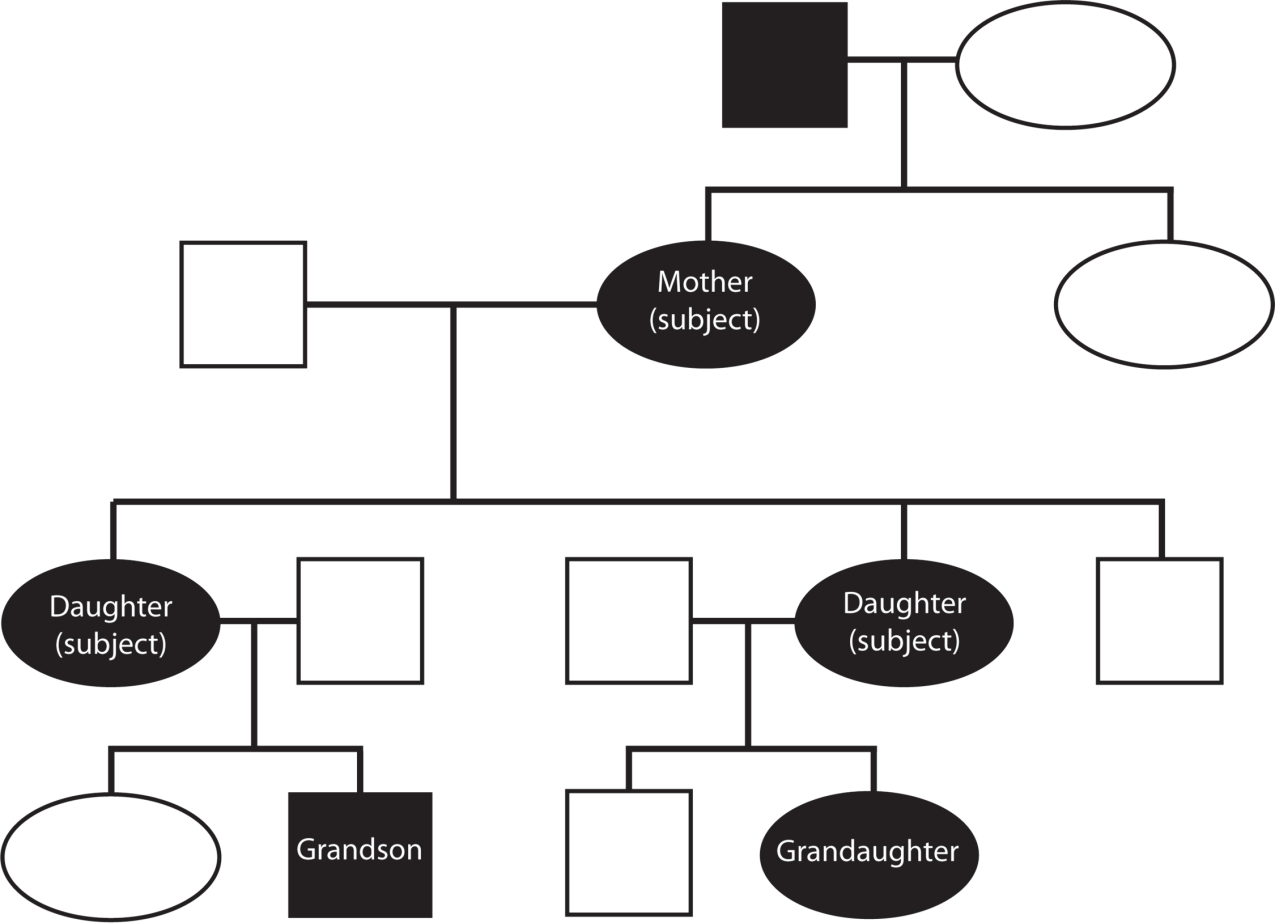
Pedigree chart illustrating the relationship of the three patients recruited. Note, two of the subjects also had children with nystagmus.

Only patients with idiopathic INS from one primary family were evaluated. The patients in the current study had good visual acuity and did not have other ocular pathologies associated with INS. The patients were screened by ophthalmological exam, patient history, ocular motility recordings and, electrophysiological testing (i.e., VER or ERGs) to ensure accurate diagnosis. A modified checklist, originally outlined by Dell’Osso [49], was undertaken with each INS patient. For example, a decelerating slow eye movement is not typical of INS. Patients with one or more coexisting conditions like optic nerve hypoplasia or retinopathy of prematurity (ROP) were also excluded because of anatomical abnormalities. INS patients needed to also have reasonably good visual acuity as a level worse than 20/70 would be suggestive of either additional pathology or lack of a null zone or minimal foveation time. INS patients needed to have a null zone for the novel procedure to work and the experimental set-up required a null zone within 15° of primary gaze. The tradeoff of a tighter inclusion criteria resulted in a lower number of participants. The three patients recruited were related, Fig 1.

Although INS is seen a few months after birth, it is not a progressive disease [50] thus, the subjects included three related females that ranged in age from 21 to 57 years, with a mean of 35 years all currently diagnosed with INS. The control group consisted of three normal females that ranged in age from 23 to 55 years, with a mean of 34.7 years. The selection process of the INS subjects was based on the following criteria: patient and family history, ophthalmological exam, eye movement studies, electrophysiology, no history of eye muscle surgery, good binocular alignment (binocular vision) and a null or semi-null zone based on patient chart review and vision tests. The control group all had no history of eye problems except for refractive error, and no known neurological problems. Fully-informed, signed consent was obtained from each subject, and the study was approved by the Institutional Review Board at Nationwide Children’s Hospital, Columbus, Ohio.

### Psychophysics

Monocular visual acuity was measured using an ETDRS visual acuity chart using a 10-alternative forced-choice procedure, as described previously [51]. Contrast sensitivity functions were measured using a Vistech 6500 (Vistech Consultants, Inc., Dayton, OH) wall-mounted, chart which utilized a 3-alternative forced choice procedure as previously described [52]. Subjects with INS undertook psychophysical tests with free head and body posture and were not constrained in any way in order to maximize visual function. The test results were used for subject selection, for descriptive purposes and to further assess any differences among INS patients (i.e., in terms of homogeneity of INS population statistics).

### DTI Scanner and Site Localization

The Magnetic Resonance (MR) scanning was performed on a 3.0 Tesla imager (General Electric Medical Systems, MR software version 15.0 with 8 channel array head coil, Brainwave software) at Nationwide Children’s Hospital. The protocol included a pre-screening brain MRI with sagittal T1-weighted and axial T2-weighted scans to exclude any anatomic brain abnormality (e.g., Chiari malformation may cause INS like nystagmus [53]).

### DTI Data Acquisition

DTI acquisition parameters were: TE = 76 ms; TR = 10 s; flip angle = 90° single-shot; full k-space; 128x128 acquisition matrix interpolated to 256x256 with a field of view (FOV) = 30 cm, which generated an in-plane resolution of 1.17 mm^2^ with full head coverage; slice thickness = 5 mm; and slice gap = 1.5 mm. Diffusion MR images were obtained from 25 directions with a b-value of 1000 s/mm^2^ along with a b = 0 image with no diffusion gradients. For anatomic imaging, a three-dimensional volume spoiled gradient-echo pulse sequence (0.469 mm^2^) in the axial plane was used and obtained 1.3 mm thick slices.

### DTI Data Analysis

Voxelwise statistical analysis of the fractional anisotropy (FA), mean diffusivity (MD), radial diffusivity (RD), first (λ1), second (λ2) and third (λ3) eigenvalues data was carried out using TBSS (Tract-Based Spatial Statistics, [54]), part of FSL (http://www.fmrib.ox.ac.uk/fsl/) [55]. FA, MD and RD were calculated using the following equations respectively where the λ’s denote the eigenvalues.

$$FA=\sqrt{\frac{1}{2}}\frac{\sqrt{\left({\left(\lambda_{1}-\lambda_{2}\right)}^{2}+{\left(\lambda_{2}-\lambda_{3}\right)}^{2}+{\left(\lambda_{3}-\lambda_{1}\right)}^{2}\right)}}{\sqrt{\left(\lambda_{1}^{2}+\lambda_{2}^{2}+\lambda_{3}^{2}\right)}}$$ 1

$$MD=\frac{\lambda_{1}+\lambda_{2}+\lambda_{3}}{3}$$ 2

$$RD=\frac{\lambda_{2}+\lambda_{3}}{2}$$ 3

First, FA images were created by fitting a tensor model to the raw diffusion data using the diffusion toolbox (FDT), and then brain-extraction was carried out using the brain extraction tool (BET) [56]. All of the subjects' FA data were then aligned into a common space using the nonlinear registration tool FNIRT [57, 58], which uses a b-spline representation of the registration warp field [59]. Next, the mean FA image was created and thinned to construct a mean FA skeleton which serves as the center of all tracts common to the group. Each subject's aligned FA data was then projected onto this skeleton, and the resulting data fed into voxelwise cross-subject statistics. This was repeated for MD, RD, λ1, λ2, and λ3. Three levels of regions of interest (ROIs) were analyzed, (1) the pons of the brainstem, (2) the cerebellum and brainstem, and (3) the cortex for statistical significance of P<0.05. In addition, Trackvis and Diffusion toolkit (www.trackvis.org) were used for the tractography, with an angle of angle of 35°, in order to quantify the number of tracks at the pons, thalamus, brainstem and right and left cerebellar hemispheres. The ROIs were selected manually. In addition to the statistical tests in TBSS, two sample t-tests with 95 percent confidence intervals, using the R-Project for Statistical Computing (http://www.r-project.org/), were performed on the number of fibers in the pons, cerebellum, and brain.

## Results

**Fig 2:**
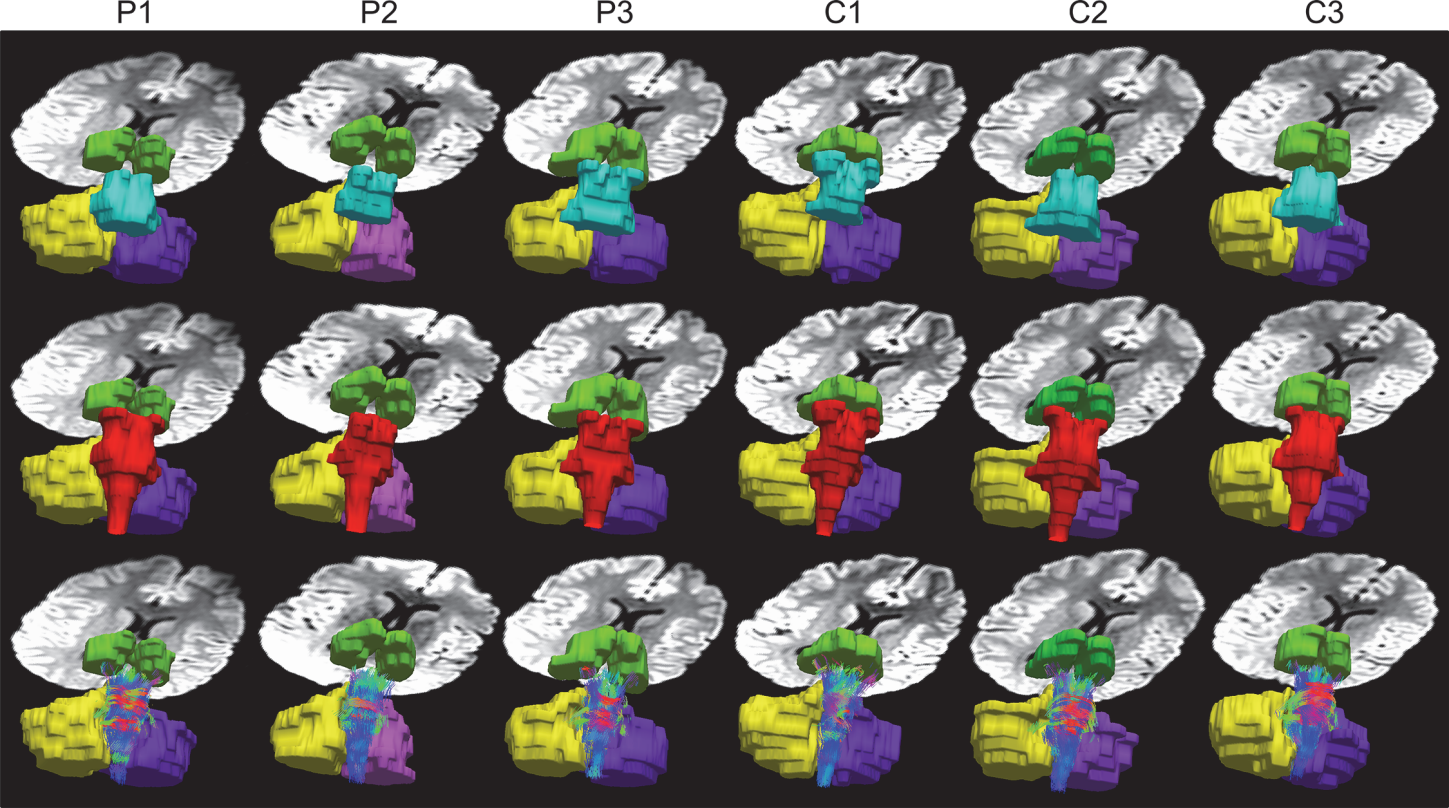
An illustration of the six ROIs (green—thalamus, blue—pons, red—brainstem, yellow—right cerebellum, purple—left cerebellum) used for quantifying number of tracks between patient and control groups. Fiber tracks in all ROIs were reduced in INS subjects (1^st^ three columns from the left) compared to normal subjects (1^st^ three columns from the right). Third row shows fiber tracks from the brainstem ROI.

**Fig 3:**
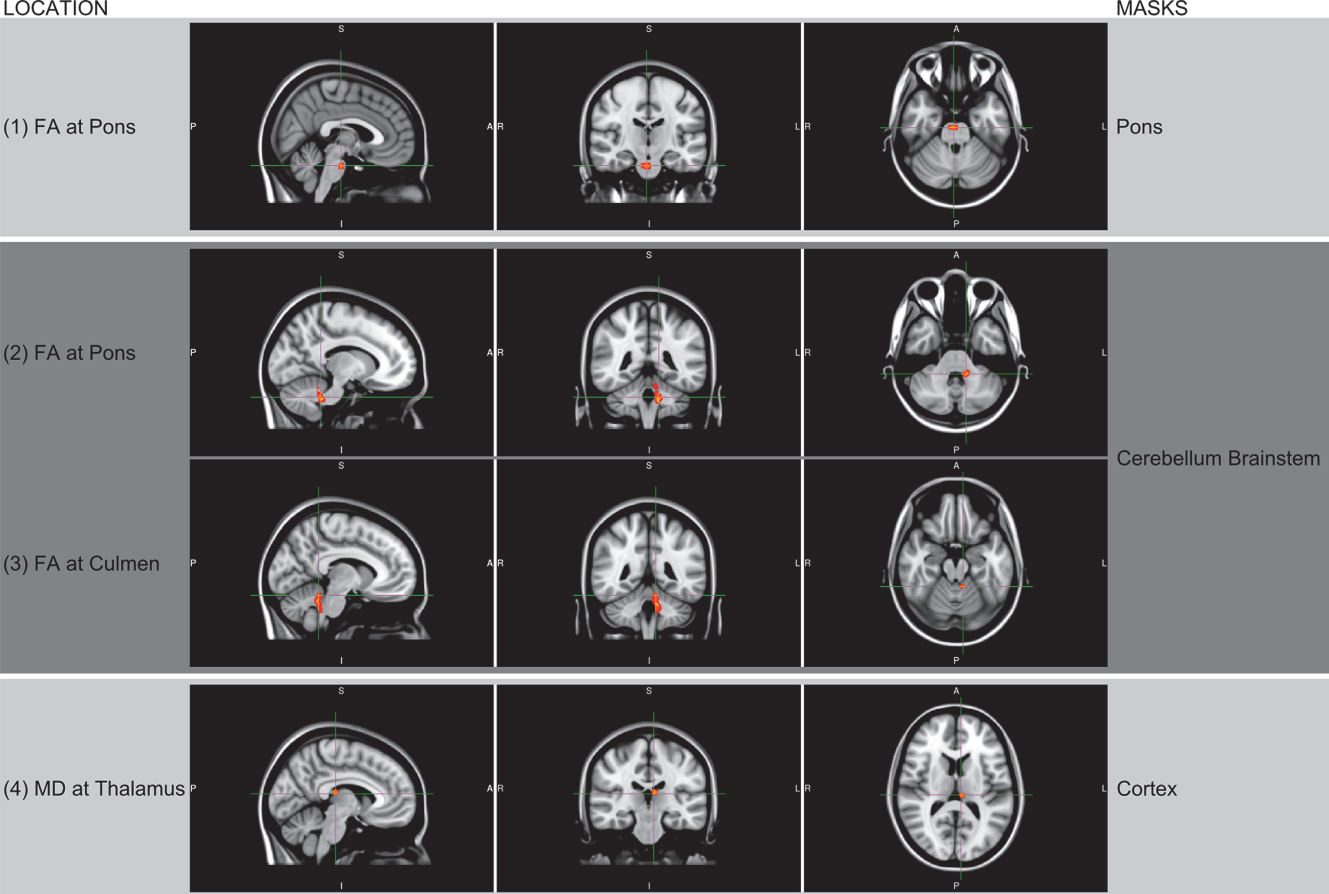
TBSS (Tract-Based Spatial Statistics) resulted in significant differences in FA values at the pons of the brainstem between the INS group and the controls (seen in orange) (N = 6) (row 1) using an ROI at the pons. FA value reduction were seen at the pons and culmen (rows 2 and 3) using an ROI at the cerebellum and brainstem for INS group. Significant reduction in MD were also seen at the thalamus (row 4) using an ROI at the cortex for INS group. Image orientation and display labels are in radiological convention. P<0.05 for all analyses.

The results of the manual ROI selections showed a reduced number of fibers for INS patients in the pons, cerebellum (right and left), brainstem, cerebrum and thalamus (Fig 2 and Table 1). Specifically, the fiber tracks across all ROIs were reduced in INS subjects, compared to normal subjects, by 15.9%, 13.9%, 9.2%, 18.6%, 5.3%, and 2.5% respectively. The TBSS results for the pons mask yielded lower FA values for the INS patients in the superior ventral aspect of the pons of the brainstem than in that of the controls (P<0.05), Fig 3. The cerebellum and brainstem mask, confirmed this finding at the pons with the addition of the culmen of the cerebellum (P<0.05). The cortex mask resulted in lower MD (P<0.05) for the patient group in the thalamus, Fig 3. The reduction in these regions was predominately on the right side. Investigation of other areas of the brain did not reveal significant differences in any of these parameters. Analyzing other parameters, such as RD, λ1, λ2 and λ3 eigenvalues did not result in any significant differences in the pons, cerebellum or the rest of the brain. A one-to-one comparison between patient and control at the individual level did not yield significant differences.

**Table 1:** Number of fiber tracks for each participant at the specified region of interest and the entire brain, where cbm-R and cbm-L are right and left cerebellum regions respectively.

**Patients**						
** **	**cbm-R**	**cbm-L**	**thalamus**	**brainstem**	**pons**	**brain**
P1	7597	8101	5246	4789	3650	144148
P2	5931	5697	4583	3405	2968	130732
P3	5954	5902	5865	4044	3771	118866
**Mean**	**6494**	**6567**	**5231**	**4079**	**3463**	**131249**
**Controls**						
C1	6911	7006	5259	5115	4266	129309
C2	7790	7566	5624	5297	4202	141260
C3	7938	7124	5214	4629	3885	145090
**Mean**	**7546**	**7232**	**5366**	**5014**	**4118**	**138553**
**Difference**	**13.9%** ^**1**^	**9.2%** ^**2**^	**2.5%** ^**3**^	**18.6%** ^**4**^	**15.9%** ^**5**^	**5.3%** ^**6**^

Two sample t-tests yielded

^1^(t = -1.6496, df = 3.213, p-value = 0.1915)

^2^(t = -0.8442, df = 2.196, p-value = 0.4805)

^3^(t = -0.3425, df = 2.485, p-value = 0.7589)

^4^(t = -2.0862, df = 2.901, p-value = 0.1313)

^5^(t = -2.3693, df = 2.847, p-value = 0.1032)

^6^ (t = -0.8383, df = 3.436, p-value = 0.4562).

## Discussion

In this study, DTI was utilized to compare the number of white matter tracks between INS patients and controls. Based on a review of the literature, this is the first study to use these imaging techniques to assess the white matter tracks of patients with nystagmus. It was hypothesized that loss of fibers at the level of the pons and cerebellum could contribute to the involuntary ocular-motor dysfunction characterized by INS. Does the decrease of the number fibers in the cerebellum hint to dysfunction in the connections? Further investigation of the cerebellum-brainstem using DTI will be crucial in identifying trouble regions in these pathways.

We also chose to investigate the white matter structure in brain with focus on the brainstem and cerebellum between INS patients and normal controls, due to previous knowledge of functional and structural connectivity of the oculomotor system, with the hypothesis that there will be differences in the brainstem-cerebellum connections (refer to [60] for detailed DTI of brainstem-cerebellar connections). The decrease in FA values in the pons and culmen may explain the involuntary eye movements seen with INS as horizontal eye movements are produced by premotor neurons in the pons. They may also be causing continuous innervation at the pons which prevents the eyes from normally being still. This is because microstimulation of neurons in the paramedian pontine reticular formation (PPRF) of pons causes horizontal eye movements [61]. Again, these are the first results to identify a decrease in FA values as possible factors for the oculomotor dysfunction associated with INS. The fact that there is an FA difference in the pons could mean a deficiency affecting the pons-declive, pons-culmen, pons-flocculus, etc. This inevitably affects the brainstem-cerebrum pathways.

The decreases of MD in the thalamus are not counterintuitive as this region is the pathway from both the cerebellum and basal ganglia to the cortex. The thalamus also connects the subcortical and cortical oculomotor centers that are responsible for the coordination of voluntary and reflexive eye movements [62, 63]. In addition, chronic lesions of the posterior thalamus have a specific oculomotor effect on the neural control of ocular fixation [62]. It is evident from our findings and the intricate connections discussed in literature that isolating and underlying anatomical correlate will need additional investigations. Hence, more extensive functional and structural studies should focus on these regions to advance our understanding of these abnormal eye movements. While little is known of the structural and functional correlates of INS; that is, the brain sites that generate the abnormal eye movements, the types of abnormal eye movements that characterize INS are well known.

Also, it is well known that normal oculomotor circuitry is located in the cerebellum-brainstem regions. The use of animal cortical electrode studies have shown that the communication between the cerebrum and cerebellum maps out through several intercalated brainstem nuclei and are heavily interconnected through the basilar PN [42]. This includes dense connections from PN to dorsal paraflocculus including petrosal lobule, moderate in the uvula and little in vermal lobuli VI-VII [64]. However, it was previously found that the densest target of the dorsolateral PN to be the posterior vermis [65]. In regards to our findings, it is expected that if there is a misrouted fiber at the brainstem level then it should also be reflected at the cerebellum level, however after TBSS that was not the case. Although, counting the number of fibers in both of these regions did show an increase. This result can be better appreciated by understanding the complexities of the fine fiber connections between the brainstem and cerebellum. These interconnections are complex and involve other nuclei in the brainstem such as the nucleus of the optic tract (NOT) and pretectal olivary nucleus (PON). The PON projects to the pons via nVII [66–70]. Both the NOT and PON receive dense retinal afferents [71–74]. The NOT receives input from the ventral lateral geniculate nucleus (LGNv), which corresponds to the region of the primate pregeniculate nucleus that forms a “cap” over the dorsal LGN [75]. There are also descending efferent projections from the NOT to the pons [76]: specifically, to the nucleus reticularis tegmenti pontis (NRTP) [75, 77], dorsolateral pontine nucleus (DLPN) [75, 78, 79] and medial pretetectal nucleus (MPN) [75], whose neurons project to a number of cerebellar regions such as the flocculus and paraflocculus [80–83] and caudal fastigial nucleus [84]. In macaque monkey, the major cerebellar target of the NRTP efferents was reported to be lobule VII of the posterior vermis [85, 86] as well as the uvula and the flocculus [87]. These animal studies have helped with localization of anatomical and functional regions, specifically in the brainstem and cerebellum, and have provided ample evidence of these connections for both normal and abnormal oculomotor function. More specifically, functional studies on normal oculomotor function have supported the animal literature in the involvement of the cerebellum. Areas of both cerebellar hemispheres including the superior semilunar, simple, quadrangular and inferior semilunar lobules have been shown in small field horizontal optokinetic nystagmus (OKN) as well as in voluntary saccadic eye movements [88]. In addition, activation was seen in the middle cerebellar peduncle, dentate nucleus, culmen (medially), and uvula of the cerebellar nuclei. We also found activation in the declive and cerebellar tonsil with OKN [48]. With ample evidence revealing a cerebellar disorder for INS patients, it can be hypothesized there is a problem with the vestibular afferents passing through the PPRF and the dorsomedial rostral medullary reticular formation and the rostral interstitial nucleus of the MLF. The vestibular nerve (nVIII) activates semicircular canals projecting the vestibular nuclei within the brainstem to the abducens nuclei, which stimulates (1) lateral rectus via nVI and (2) MLF via nIII to medial rectus muscle. Since the normal vestibulocerebellar function rebalances the VOR, any unwanted nystagmus occurrences are corrected. With permanent damage to the vestibular afferents, there is no recorrection and the eye drifts are maintained. The plasticity of a normal brain would be able to recognize these oculomotor changes for correction. Unlike vertical reflex signals, horizontal reflex signals have more unknowns. Inhibitory tonic vestibular pause (TVP) signals are seen on vestibular nuclei cells in the abducens nucleus during these reflexes; however, lesions of the vestibular nuclei does not abolish horizontal eye movements so focus on the nVI pathway is more crucial in future studies. Furthermore, fMRI studies on oculomotor dysfunction have been in agreement with the animal literature. fMRI was used to investigate downward smooth pursuit eye movements versus straight-ahead fixation of a stationary target in controls and in patients with idiopathic downbeat nystagmus (DBN) at 1.5 Tesla [89]. This data revealed reduced activation in the parafloccular lobule and ponto-medullary brainstem of the patients. Another group [90] studied DBN with smooth pursuit in both downward and upward directions and found differences in the floccular lobes. Fastigial nucleus has also been reported to be active in opsoclonus [91]. More recently, our research [27] showed fMRI activation in the declive and uvula in INS patients at 3 Tesla (note: these were the same participants as in this study). With respect to both structural and functional studies, other than our findings, no animal lesion studies or human clinical studies have uncovered the anatomical correlates of the INS. All these studies have some activation in the cerebellum but are meager in the brainstem mainly because of fMRI imaging limitations. Naturally, based on these functional studies we can test the structure connectivity. Thus our goal was to identify the structural connectivity sites that may be associated INS. The reason we were not able to fully differentiate the fine pathways seen in the animal studies could be a combination of factors. One is that the skeleton model produced may not cover all the pathways and is dependent on a user controlled threshold. The other factor is the imaging resolution. The latter affects both the FA maps generated as well as the tractography, i.e. fiber track counts. Also, the resolution influences the accuracy of the manual selection of the ROI at the pons and cerebellum for the tractography data. There are common crossing and termination problems in regards to the fiber tracts with lower magnetic field strengths as well. However, line propagation algorithms are able to capture the directions and degrees of crossing fibers at higher resolution and field strength. Furthermore, DTI has limitations regarding the size of a single white matter voxel. With larger voxels, the probability of containing multiple fiber bundles with different directions increases. A more comprehensive analysis of the brainstem and cerebellum that would include higher resolution, more participants and further correlation with fMRI would be a good second stage to this study. Although not an issue for our current study population, another possible confound that we would need to overcome in the future is that INS patients may represent a heterogeneous population, with different and multiple INS sites. This issue can be addressed by screening INS patients for any related pathology (e.g., albinism) or other abnormal eye movements such as Periodic Alternating Nystagmus (PAN), by requiring visual acuity of 20/70 or better and by meeting the requirements of a comprehensive inclusion/exclusion check list.

Additional explanation of our results can be attributed to the fact that DTI is more sensitive to white matter as compared to gray matter structures. Therefore, the finding in the thalamus may indeed be a false positive. In particular the decrease in MD is normally not considered abnormality for water diffusion in the brain. In general decrease in FA is associated with increase in MD however we report a decrease for the FA in the pons while there was no associated RD or MD differences. These results in general do not follow the majority of studies on abnormalities of water diffusion reported in the human brain. These findings are affected by the choice of large slice thickness and using a gap produce suboptimal images when studying small structures such as brain stem, pons and thalamus. This was a necessary tradeoff in order to minimize inhomogeneity artifacts and increase the signal to noise ratio (SNR). Further investigation of these areas will contain optimized imaging protocols. Overall, these results are a significant initial phase for further analysis of these intricate connections between the brainstem and cerebellum. This will allow future work to expand these findings to all other oculomotor dysfunction, such as other types of nystagmus and gaze-holding disorders, including those caused by traumatic brain injury. Thus, our research would be the first step in the development of long term drug or surgical treatments to improve vision and quality of life for patients who suffer from nystagmus. Indeed, the combination of both functional and structural imaging has proven to be of great benefit, whether a specific pathway [92, 93] is of interest or a particular clinical study, such as preoperative imaging for tumor delineation, is needed [94, 95]. Combining fiber tracking and functional activation will result in enhanced determination of the relevant sites associated with INS.

## Conclusion

We have identified some brain regions that may be actively involved in INS. These novel findings would be beneficial to the neuroimaging clinical and research community as they will give them new direction in further pursuing neurological studies related to oculomotor function and provide a rational approach to studying INS. This new knowledge as a result can be applied across other diseases with oculomotor dysfunction, such as other types of nystagmus and gaze-holding disorders, including those caused by traumatic brain injury. More importantly this will improve the quality of life for all persons and families affected by INS. The presence of a decrease in the FA, at the pons, culmen and decrease in the MD at the thalamus, for the INS group, could mean a deficiency affecting any of the following pathways: pons-declive, pons-culmen, pons-flocculus. This inevitably affects the brainstem-cerebrum pathways. Furthermore, the loss of fibers in both cerebellar hemispheres, the brainstem and the pons in patients with a horizontal nystagmus leads to predictions of the primary cause being abducens and oculomotor nerve lesions. Future functional and structural studies with more optimal resolution should focus on these regions to advance our understanding.
